# Development and characterization of biparental mapping population for the properties of seed and seed coat applicable to seed longevity in soybean (*Glycine max* (L.) Merrill)

**DOI:** 10.1186/s12870-025-08018-z

**Published:** 2025-12-27

**Authors:** R L Ravikumar, Naflath Thenveettil, Mugali Pundlik Kalpana, A Hemanth Kumar, Athulya S Nair, B S Patil

**Affiliations:** 1 Department of Plant Biotechnology, College of Agriculture, UAS, GKVK, Bangalore, Karnataka 560 065 India; 2 Department of Seed Science and Technology, College of Agriculture, UAS, GKVK, Bangalore, Karnataka 560 065 India; 3 ICAR-IARI Regional Research Centre, Dharwad, Karnataka 580 005 India; 4 Department of Plant and Soil Sciences, Mississippi State University, Mississippi State, MS 39762 USA; 5 ICAR-Indian Institute of Agricultural Biotechnology, Ranchi, Jharkhand 834003 India

**Keywords:** Seed viability, Recombinant inbred lines, Seed coat colour, Seed coat thickness, Seed permeability, Mapping population, Seed logevity

## Abstract

**Background:**

Short life span of soybean seed under ambient storage conditions is a serious challenge for crop production and germplasm maintenance. There are scarce reports on linkage mapping of QTLs for seed longevity, and the molecular mechanisms remain largely unclear. The study aimed to develop a biparental recombinant inbred lines (RIL) mapping population suitable to examine seed and seed coat properties contributing to seed viability as well as seed yield and yield traits. An RIL population from JS 93 − 05 × Local Black Soybean (LBS) cross was developed and evaluated for seed and seed coat properties at F_5_ generation providing a robust resource for QTL mapping and breeding for improved seed longevity.

**Results:**

Comprehensive assessment of RIL population for seven seed and seed coat properties including water permeability and seedling vigor index revealed broad and continuous variation in quantitative traits. The mean, range and coefficient of variation of these 7 seed traits indicated the presence of high variability in the RIL population. Distinct segregation in qualitative seed coat features, such as seed coat colour, hilum colour and seed coat lustres further underscore the involvement of these traits on seed longevity. RILs with black seed coats exhibited an association between higher seed coat content and reduced water permeability, suggesting pigmentation mediated enhanced seed longevity. Furthermore, RIL lines with low-permeability displayed greater seed coat thickness, reinforcing the role of physical seed coat traits in regulating water uptake.

**Conclusions:**

The developed biparental F_5_ mapping population demonstrates extensive genetic and phenotypic diversity for seed and seed coat traits, offering a valuable resource for mapping loci associated with seed longevity.

## Background

Soybean (*Glycine max* [L.] Merr.) is a leading oilseed crop grown in many countries with seeds containing approximately 40% protein, 20% oil, and 30% carbohydrate, making them highly valuable for food, feed, and processing applications [1, 2]. In 2024, India ranked fourth in area with 13.26 million hectares, accounting for 9.50% of the global cultivated area, and fifth in production with 13.06 million tonnes [3], and the demand for soybean continues to increase world-wide including India. Soybean area for India depicts an overall upward trend in recent years [4]. However, soybeans are associated with poor seed longevity [5]. During storage, soybean seeds experience a decline in quality, viability and a reduced germination capacity, ultimately leading to significant yield losses in soybean crop. The short life span of soybean seed (< 8 months in tropics) under ambient storage conditions hampers its usage in the ensuing seasons by farmers thus burdening the seed industry with entire seed supply [6]. In cultivated crops, extended seed longevity is important for retention of seed germination and seed vigor during storage for meeting the norms of regulatory regimes and profitable seed production. The phenomenon of seed deterioration involves numerous physical, biochemical, and physiological changes, including the loss of enzymatic activities and membrane integrity, and genetic alterations [7–14]. However, the mechanisms by which the aging process affect soybean seed viability have not yet been fully elucidated [15].

Substantial efforts have been made to understand the potential causes for the deterioration of viability in soybeans and in other crops [16]. Seed coat is an important protective agent that shields the underlying embryo from quick deterioration. The degree of protection offered by seed coat depends on its structural properties of the seed surface, such as cutin deposition, presence of cracks, and the arrangement of other chemical deposits on the seed surface, all of which influence the permeability, thus longevity [17, 18]. Generally, high seed permeability is associated with rapid deterioration of lignin content and seed longevity [19, 20]. The size, shape and chemical deposition of the testa is also correlated with permeability [21–23]. The black or dark seed coat colour genotypes with a few pores on the seed coat, small seed size, and high lignin content prolong seed longevity in soybean [24–27]. Higher proportion of seed coat and seed coat thickness also showed positive association with viability in soybean [18, 28]. In summary, the properties of seeds like seed size, seed weight, seed coat thickness, seed coat colour, seed surface properties, hilum colour, and water permeability are the primary factors influencing viability in soybean.

The advances in molecular mapping technologies enable QTL and gene mapping, which enables breeders to identify, select, and combine desirable traits to improve crop varieties by pinpointing genome regions responsible for natural phenotypic variation in complex traits [29, 30]. Biparental mapping population offers simplicity and the ability to control the genetic background [31] but are limited by the number of allelic variants per locus that can be evaluated [32]. There are scarce reports related to the linkage mapping of QTLs for soybean seed longevity, and the molecular mechanisms underlying soybean seed longevity are largely unclear. However, a few studies using biparental mapping population have identified QTLs for seed longevity in soybean [21, 33, 34] which were not repeatable in different mapping populations [35]. Singh et al. [33] identified four SSR markers (*Satt434*,* Satt538*,* Satt281*,* and Satt598*) explaining 6.3 to 7.5% of variation for longevity. While Hosamani et al. [21] reported linkage of three SSR markers with testa color and seed storability. In one study, QTL mapping conducted on F_2:3_ and F_3:4_ seeds under both natural and artificial ageing conditions identified 13 SSR markers distributed across six linkage groups [36]. Zhang et al. [34] performed a more extensive analysis using two RIL populations and high-density SNP linkage maps, uncovering 34 QTLs located on 11 chromosomes, including two hotspots on chromosome 5 and 17. A recent genome-wide association study by Thenveettil et al. [37] revealed 28 candidate genes associated with seed longevity in soybean. Genes associated with late embryogenesis abundant proteins, heat shock proteins, transcription factors, RNA helicase, delay of germination (DOG-1) domain proteins, GIBBERELLIN 2-OXIDASE 1 (GA2OX1), ENHANCED RESPONSE TO ABA1 (ERA1), ETHYLENE INSENSITIVE 2 (EIN 2) [37–40] are among the many candidate genes that were reported to be associated with seed longevity. These existing studies have reported diverse and inconsistent genetic control of seed longevity in soybeans. Successful improvement of seed longevity depends in identifying genomic loci that contribute significantly to phenotypic variation, along with key physiological and physical seed traits associated with it. These seed traits would render easy identification of superior sources for seed longevity.

The diversity and recombination in the mapping population are essential in identification of markers closely linked to the trait [41]. The choice of the parental lines and the mapping population for the trait of interest, size of the mapping population and the density of the markers determine the stability of the markers identified for the QTL. Until now, there were only a few findings focusing on the mapping of QTL for the properties of seed coat, seed size, shape and permeability of seeds in soybean [42]. Despite the more sophisticated methods which are now available, linkage maps remain important for QTL analysis [43]. Considering this, we have developed a large biparental RIL population suitable to examine seed and seed coat properties contributing to seed viability. A large number of RILs is crucial for higher statistical power to detect QTLs, better accuracy in determining the effect and higher resolution mapping [44]. This study describes the development and initial characterization of the RIL population for seed coat and seed characteristics relevant to seed viability in soybean. It is based on the hypothesis that seed longevity is influenced by both seed and seed coat properties [25]. Our findings represent a valuable reference population and a resource for the future research on soybean seed viability as it encompasses diverse allelic variation for traits governing seed longevity.

## Methods

### Hybridization of parental lines

We developed a biparental mapping population by crossing JS 93 − 05 with Local Black Soybean (LBS) during kharif season of 2020. JS 93 − 05 is a commercial variety developed by Jawaharlal Nehru Krishi Vishwavidyalaya, Jabalpur and the male parent Local Black Soybean (LBS) was collected at University of Agricultural Sciences, Dharwad and maintained by the first author for the use in research. The female parent is a yellow seed coat colour genotype with bold seeds (seed weight: 13.8 g/100 seeds). It has determinate, semi-erect growth habit, lanceolate leaf shape, purple flower colour and brown pods. The male parent is a black seed coat colour genotype with medium seed size (seed weight: 7.98 g/100 seed). It has indeterminate, semi-erect growth habit, pointed ovate leaf shape, purple flower colour and yellow colour pod. The parental lines were evaluated for seed longevity using accelerated ageing (AA) method [45]. During AA, the seeds were subjected to 41 ± 0.3 °C temperature and more than 95% relative humidity in an ageing chamber for 72 h. The female parent exhibited only 6% seed germination, while LBS showed 85% germination after AA [46]. The parental lines also differed for various agronomic and yield related traits [46]. The two genotypes were grown in pots in the greenhouse during kharif 2020 and F_1_ from the cross was produced. The F_1_ plants were grown during kharif 2021 and the true F_1_ plants were identified by carefully observing the morphological traits of individual F_1_ plants which were later confirmed using DNA markers [46]. The F_1_ progenies were then selfed under controlled conditions to produce F_2_ progenies. A total of 352 F_2_ seeds were obtained from true F_1_ plants.

### Advancement of F_2_ population to F_5_ generation recombinant inbred lines

Three hundred and fifty-two F_2_ seeds harvested from true F_1_ plants were grown in 16 rows with 22 plants per row during *Rabi*, 2021. A row of parental lines was sown after every 5 rows of F_2_. A spacing of 40 × 15 cm was maintained between rows and plants, respectively, to avoid competition between the plants. All the agronomic practices were followed to raise a good crop. Subsequent cycles of controlled selfing were conducted for each F_2_ plant, ultimately producing F_5_ generation seeds by 2024. The inbreeding phase utilized a single seed descent (SSD) strategy, where the seeds of one plant were grown and only one plant was harvested per line and advanced to succeeding generations. Totally F_5_ generation seeds produced from 352 lines during Kharif 2024 were used for advancing to succeeding generations as mentioned earlier. The following observations were recorded on F_5_ generation RIL population.

### Seed and seed coat characteristics

For the present study, the row bulk of F_5_ generation seeds were used. Out of 352 lines, a representative 150 lines were chosen randomly for recording the following observations on seed characteristics. The observations on three qualitative seed characteristics, namely seed coat color, seed hilum color, and seed coat lustre were recorded in five replications based on manual observation according to the soybean crop descriptor [47]. In the present study using 150 RILs, the seed coat colour was broadly represented by four colours, black, yellow, yellow green and green; the seed hilum colour was represented by grey, brown, brownish black/imperfect black, and black; and the seed lustre by shiny, intermediate, dull, and bloom. The quantitative variables measured are seed length, seed width, seed thickness, seed coat percentage, water permeability, seedling vigour, 100-seed weight and seed coat thickness. The length of the seed was measured as the longest distance across the seed parallel to the hilum, the seed width as the longest distance from top (hilum) to bottom of the seed, and the seed thickness as the longest distance across the seed perpendicular to the hilum on five randomly selected seeds from each RIL genotype [48]. To measure the seed coat percentage, ten seeds per RIL per replication (3 replications per RIL) were randomly drawn and the seeds were soaked in water for 24 h after which the seed coat was carefully peeled from the cotyledons. Both seed coats and cotyledons were then dried in a hot air oven at 90 °C for 48 h. After drying, the weights of the seed coat and cotyledons were recorded, and seed coat percentage was estimated as follows:

$$\text{Seed coat }\left(\%\right)=\frac{\text{Seed coat weight}\left(\mathrm{mg}\right)}{\text{Seed coat weight} \left(\mathrm{mg}\right)+\text{Cotyledon weight}\left(\mathrm{mg}\right)}\times100$$

The water permeability of the seed coat of all the 150 RILs was checked after 3 h of soaking in water in petri plates (rapid imbibition). Based on the preliminary study, 3 h of rapid imbibition was sufficient to discriminate the RILs for seed coat permeability (data not given). Ten seeds per RIL per replication and three replications per RIL were used. The initial weight of ten seeds before imbibition and final weight after imbibition was recorded. Water permeability was quantified as the amount of water imbibed by seeds, estimated as percent water absorption.

$$ \text{Percent water imbibed}=\frac{\text{Weight of the seeds after imbibition-Initial weight of the seeds}}{\text{Initital weight of the seeds}}\times100$$

### Seedling vigor index

The seedling vigour index was estimated based on germination percentage and seedling length. The germination test was conducted using the Between paper method [49] with four replications of 50 seeds for each RIL, incubated at 28 °C. The paper towels were wetted every alternate day with water. On the 5th day, the germination percentage was determined as the (number of seeds germinated on the 5th day/total number of seeds sown) × 100. After taking the germination percentage, five seedlings from each replication were randomly chosen, and the total seedling length was measured in cm. The seedling vigour index was calculated using the following formula,

$$\text{Seedling vigour index}=\text{Germiantion percentage}\times\text{Seeding length}\left(\mathrm{cm}\right)$$

Test weight for each RIL was determined as the weight of 100 seeds and expressed in grams. The measurement was carried out in three replications. To know the relationship between water permeability and seed coat thickness, ten RILs with lowest water permeability and ten RILs with highest water permeability were chosen from 150 RILs studied. The seed coat thickness of chosen 20 RILs were recorded by carefully removing the seed coat after breaking the dry seed and the thickness was measured using a screw gauge. Since the seed thickness was not constant across the seed, we have chosen two regions of the seed- top (near the hilum) and basal region of the seed considered separately for measuring seed coat thickness. Five seeds per RIL were used for recording the observation on seed coat thickness.

### Statistical analysis

Summary statistics for each quantitative trait in the RIL population was computed using MS Excel. The distribution of quantitative traits among the RILs was tested using the single-sample Kolmogorov–Smirnov (KS) test / Lilliefors test [50] assuming that the traits under study are normally distributed. The traits showed normal distribution. The RILs were grouped based on seed coat colour and significance of differences in the mean values of colour groups for different traits was tested using Tukey’s Honestly Significant Difference (HSD) test at α = 0.05 using the R software, assuming that the observations being tested are independent within and among the groups under consideration. A two-sample t-test with unequal variances was performed in MS Excel to assess the significance of differences in water permeability and seed coat thickness between low and high permeable genotypes. Pearson’s correlation between water permeability and seed coat thickness was estimated using MS Excel.

## Results

**Fig. 1:**
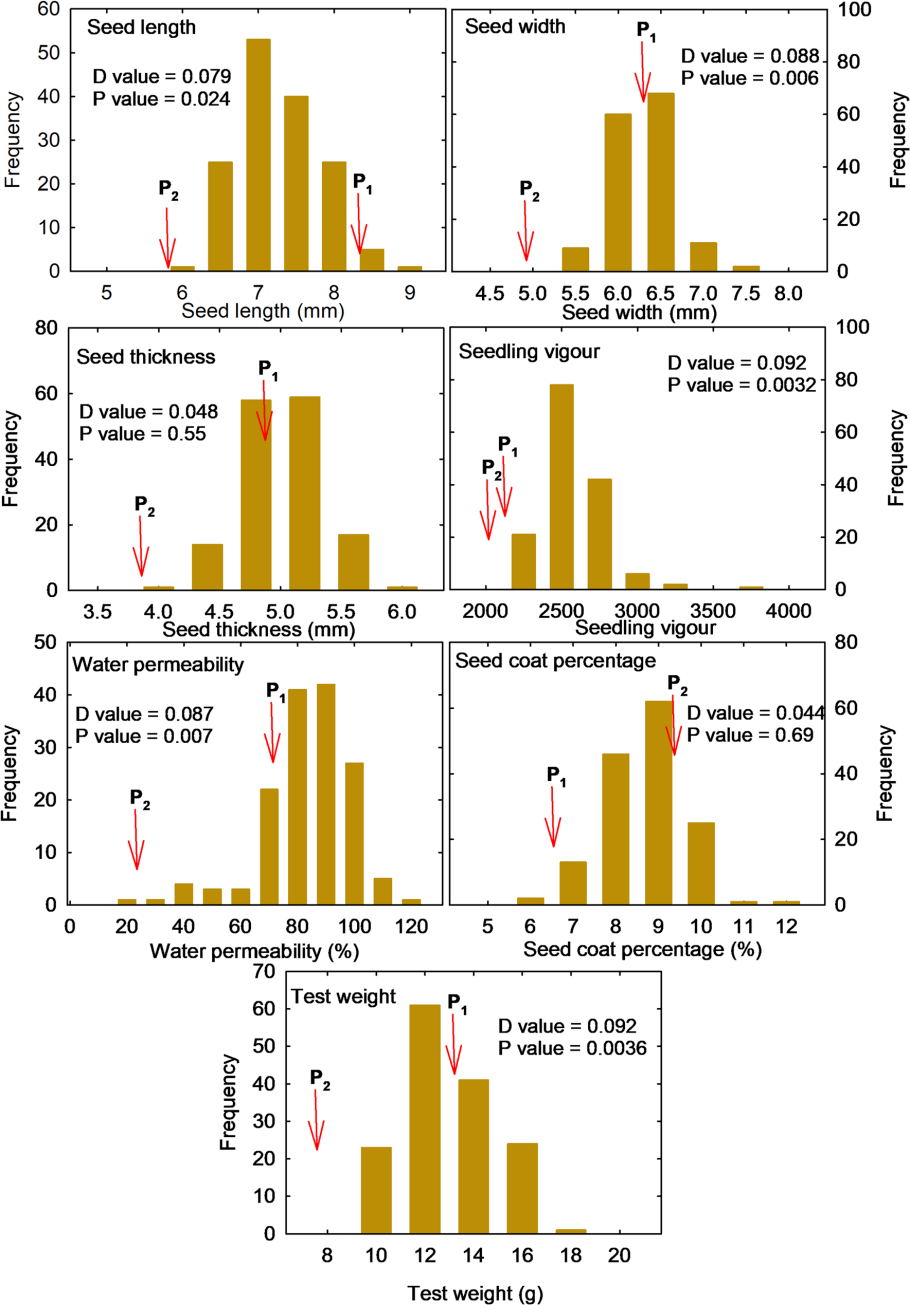
Histogram depicting distribution of RIL genotypes for quantitative traits (Note: P_1_- JS 93 − 05, P_2_- LBS). The D and P values are from the Kolmogorov-Smirnov (KS) test for normality distribution. D represents the maximum deviation between the empirical and theoretical distribution and P value is the probability value indicating whether the deviation is statistically significant

Seed and seed coat properties are important for seed longevity in soybean. The mean, range, and coefficient of variation of 150 RILs for seven seed properties such as seed length, seed width, seed thickness, seed coat percentage, 100 seed weight, water permeability, and seedling vigour are presented in Table 1. The mean seed length among RILs was 7.02 mm and it ranged from 2.69 to 8.57 mm. Seed width values in the RIL population ranged between 5.13 mm and 7.05 mm with a mean of 6.04 mm. The parental line JS 93 − 05 had higher value for seed length, seed width and seed thickness than those of LBS. The seed thickness ranged from 3.97 to 5.76 mm among the RILs population with a mean of 4.80 mm. The parental line JS 93 − 05 recorded a seed coat content of 6.99%, while LBS showed 9.39% of the seed coat content. Among the 150 RILs, the seed coat content ranged from 5.80 to 11.56%. On the other hand, the parental line JS 93 − 05 exhibited higher values than LBS for 100- seed weight, water permeability, and seedling vigour index. The mean 100 seed weight was 13.81 g for JS 93 − 05 and 7.98 g for LBS, while it ranged from 8.70 to 17.70 g across the RILs population, with an average of 12.01 g. The water permeability ranged from 15.83 to 110.79% among the RILs population with a mean of 78.06%. The seedling vigour index also showed a wide variation among the RILs with a mean of 2452.32 and it ranged from 2009.69 to 3561.73. High coefficient of variability was observed for all the traits in RILs population. The highest coefficient of variability was observed for water permeability (20.33%) followed by seed coat percentage (11.22%) and seedling vigour index (8.77%). Histograms showing the distribution of the phenotypic variation for the seven quantitative traits studied in the 150 RILs population are presented in Fig. 1, indicating a continuous distribution. A small value of D observed for all the traits indicate that the observed distributions follow the theoretical distribution. The higher P- values for all the traits suggest that the data fit the normal distribution. The Lilliefors normality test concluded normal distributions for all traits. It also illustrated that the frequency distribution varied widely, and a few outliers were also observed for all the traits suggesting transgenic segregation for all the traits.

**Table 1:** Mean, range, coefficient of variation of 7 quantitative traits in the RILs population and parental genotypes

Sl. No.	Trait	Generation	Range	Mean ± SE	Coefficient of variation (%)
1.	Seed length (mm)	P_1_*	7.97–8.83	8.34 ± 0.15	3.79
		P_2_*	5.82–6.11	5.93 ± 0.05	1.79
		RILs	2.69–8.57	7.02 ± 0.04	7.60
2.	Seed width (mm)	P_1_	6.08–6.70	6.41 ± 0.11	3.81
		P_2_	4.79–5.22	4.98 ± 0.07	3.35
		RILs	5.13–7.05	6.04 ± 0.02	5.96
3.	Seed thickness (mm)	P_1_	4.83–5.18	4.98 ± 0.062	2.82
		P_2_	3.70–4.38	3.92 ± 0.12	6.81
		RILs	3.97–5.76	4.80 ± 0.03	7.20
4.	Seedling vigour index	P_1_	1981.56-2327.04	2197.03 ± 80.03	7.28
		P_2_	1700.68–2267.00	2007.87 ± 121.15	12.07
		RILs	2009.69-3561.73	2452.32 + 17.56	8.77
5.	Water permeability (%)	P_1_	68.75–76.40	72.06 ± 2.27	5.46
		P_2_	24.32–30.64	27.24 ± 1.84	11.70
		RILs	15.82-110.79	78.06 ± 1.30	20.33
6.	Seed coat percentage	P_1_	6.90–7.13	6.99 ± 0.073	1.81
		P_2_	9.18–9.70	9.39 ± 0.16	2.90
		RILs	5.80-11.56	8.16 ± 0.07	11.22
7.	100 seed weight (g)	P_1_	-	13.81	-
		P_2_	-	7.98	-
		RILs	8.20–17.70	12.01 ± 0.16	16.61

*P_1_ = JS 93 − 05, P_2_ = Local black soybean

**Fig. 2:**
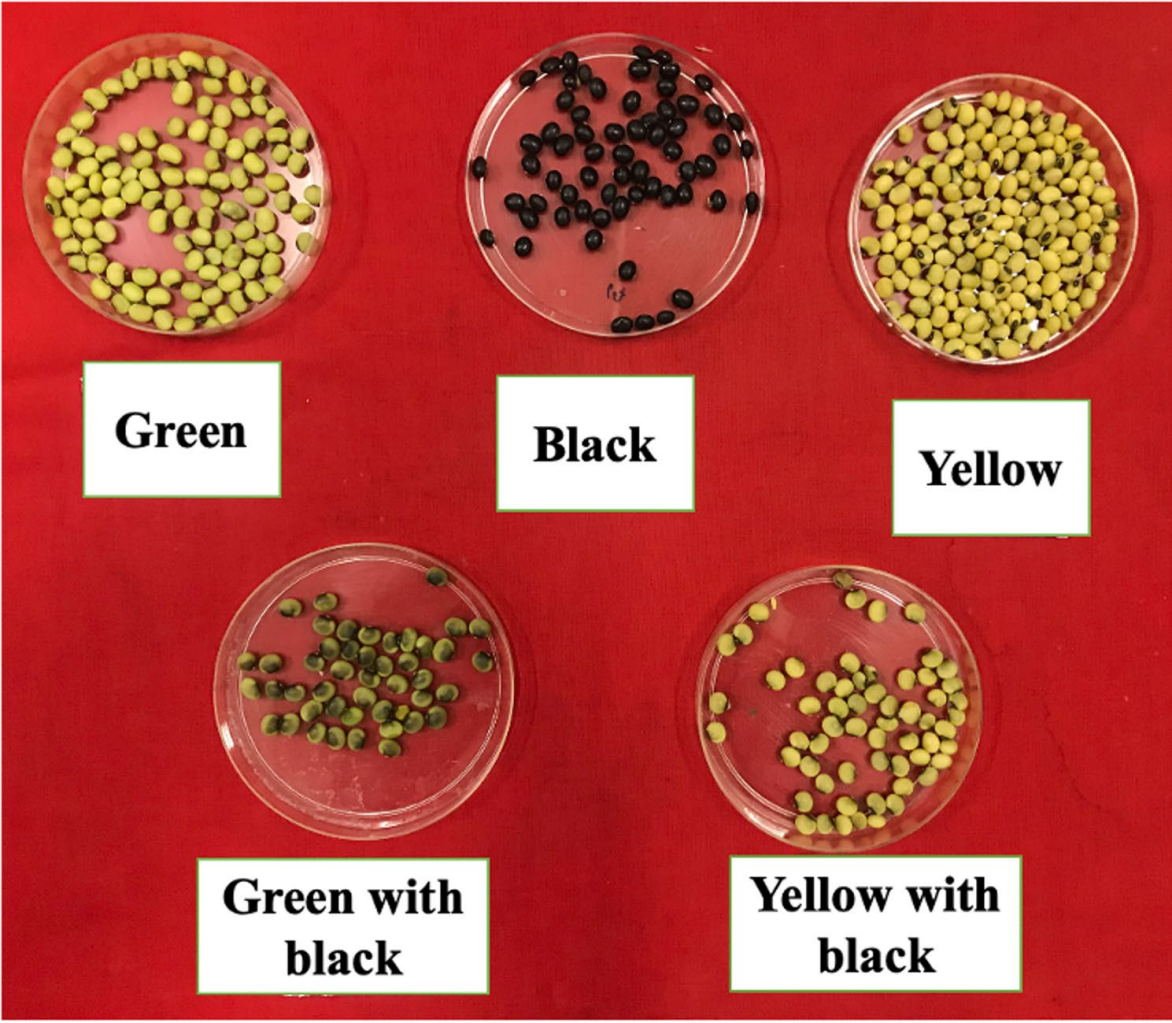
Segregation of seed coat color in the RIL population of JS 93 − 05 × Local black soybean

Three qualitative traits viz., seed coat colour, hilum colour and seed coat lustre were studied in the RILs population. The seed coat colour in the RILs could be broadly classified into 4 distinct colours: black, yellow, green, and yellow green in the RILs (Fig. 2). The highest number of RILs recorded was black seed coat colour (80) followed by yellow (30) (Table 2). The lowest number of RILs recorded yellow green seed coat colour. However, by careful observation it was noticed that within each class there were variation in the intensity of colour among RILs and also within the line. Similar variation was noticed for the hilum colour and seed coat lustre. Therefore, we did not attempt to fit the genetic ratio to study the role of gene interaction in the expression of these traits. Both the parental lines had black hilum colour. However, segregation for hilum colour was observed with 4 distinct colours. The majority of the RILs showed black hilum colour followed by imperfect black and a few RILs with grey and brown hilum colours (Table 2). Four different categories were observed for seed coat lustres. The highest number of RILs (69) had intermediate lustre followed by shiny (36) and dull (30).

**Table 2:** Segregation pattern of seed coat color, hilum colour and seed coat lustre in the RILs population

Trait	Class	Number of RILs
Seed coat colour	Yellow	30
	Yellow green	18
	Green	22
	Black	80
Hilum colour	Grey	8
	Brown	3
	Brownish black/imperfect black	15
	Black	124
Seed coat lustre	Shiny	36
	Intermediate	69
	Dull	30
	Bloom	15

The RILs were classified into four seed coat colour groups-black, yellow, yellow-green, and green, and the mean and range of seven quantitative traits were determined and compared for each colour group (Table 3). For seed length, seed width, seed thickness, seedling vigour index, and 100-seed weight, the mean values of the four colour groups did not differ significantly. However, the black colour RILs class had significantly higher mean value for seed coat percentage (8.50%) with a wider range (6.13–11.56%) compared to the other three colour groups among the RILs (Table 3). The mean values of the green (7.68%), yellow green (7.72%) and yellow (7.89%) were not significantly different. Similarly, the black seed colour RILs recorded significantly lower mean water permeability (71.06%) with a wider range of 15.83 to 110.79%. The other three classes of RILs (yellow, yellow green, and green) had significantly higher mean water permeability compared to black class; they did not differ significantly for water permeability among themselves. In conclusion the black seed coat colour class had significantly higher seed coat content and lowest permeability.

**Table 3:** Grouping of RILs based on seed coat colour and the group mean and range

Seed coat colour	No.of lines	Seed length (mm)		Seed width (mm)		Seed thickness (mm)		Seed coat percentage (%)		Water permeablity (%)		Seedling vigour index		Test weight (g)	
		Mean	Range	Mean	Range	Mean	Range	Mean	Range	Mean	Range	Mean	Range	Mean	Range
Black	80	7.08^a^	6.00- 8.15	6.01^a^	5.17–6.84	4.74^a^	3.97 - 5.60	8.50^a^	6.13–11.56	71.06^a^	15.83-110.79	2467^a^	2023–3562	11.89^a^	8.30–16.00
Yellow	30	6.99^a^	5.87–8.57	6.10^a^	5.13–7.05	4.87^a^	4.00 − 5.46	7.89^b^	6.10–9.65	88.42^b^	67.90-106.20	2456^a^	2047–3180	12.11^a^	8.20–17.70
Yellow green	18	6.99^a^	6.28–7.87	6.11^a^	5.53–7.02	4.94^a^	4.34 - 5.59	7.72^b^	5.90–9.15	83.95^b^	51.65–97.11	2445^a^	2010–2873	12.96^a^	10.20–15.90
Green	22	6.90^a^	6.29–7.97	6.04^a^	5.51–6.80	4.82^ab^	4.15 - 5.76	7.68^b^	5.80–9.83	84.78^b^	20.99–101.70	2401^a^	2108–2747	11.57^a^	8.70–15.80

Mean Values with the same superscript do not differe each other

To identify the relationship between seed coat thickness and permeability, ten RILs with highest permeability and 10 RILs with lowest seed permeability were chosen from 150 RILs. The seed coat thickness was not uniform across different regions of the seed; therefore, two regions were chosen for measuring the seed coat thickness. It was observed that the thickness of the top end of the seed (hilum region) had higher mean value compared to basal end in both the high water permeable and low water permeable RILs (Table 4). The mean thickness of the low water permeable RILs was significantly higher than that of the mean of the high water permeable RILs at both the top and bottom regions of the seed. The correlation between water permeability and seed coat thickness at the top end of the seed was significantly negative among the chosen 20 genotypes.

**Table 4:** Relationship between water permeability and seed coat thickness in low and high permeable RIL genotypes

Water permeability	Water permeability (%)	Seed coat thickness (mm)	
		Top side (hilum)	Bottom side
Low permeable	36.46^a^	0.1191^a^	0.0676^a^
High permeable	101.72^b^	0.0977^b^	0.0586^b^
Correlation between water permeability and seed coat thickness		-0.51 (p value- 0.023)	-0.41 (p value- 0.071)

Mean values with the same superscript do not differ each other

## Discussion

Seed longevity is a vital trait in soybeans to maintain continuous supply chain with high quality seeds [6]. Reduced seed longevity can lead to unexpected declines in viability during storage, thereby hampering seedling establishment and ultimately reducing crop yield [34]. Linkage analysis to dissect seed longevity in a bi-parental cross has been reported in soybean [34, 39] and other crops [51, 52]. However, the resolution and accuracy of QTL depend more on the size of the mapping population [53]. The population with high number of individuals improve the accuracy and reliability of QTL mapping [44, 54]. Majority of the earlier studies on QTL mapping in soybean are based on limited population size; therefore, no common QTLs were detected in the RILs [33, 38, 55, 56]. The most important step to discover the genetic determinants of seed longevity is development of a suitable large mapping population. In this study, two genotypes showing contrasting seed longevity, JS 93 − 05 (poor longevity) and LBS (superior longevity) were used to develop a large RIL population segregating for seed longevity and related traits [46]. The choice of the present mapping population with 352 RILs offer to improve the accuracy of QTL mapping for seed traits in the future. A large number of RILs have been collected for the future research on longevity in durum wheat [57] and rice [44, 58]. The present study focuses on the characterization of a RIL population (F_5_) with the aim of enhancing the understanding of factors influencing seed viability in future research.

Seed viability is quite complicated, influenced by many seed and seed coat related characteristics [28, 59–61]. Several findings have reported that large seed size contribute to better seed germination and seedling vigor [62, 63], while it is negatively correlated with seed longevity under storage [64]. It is mainly attributed to the increased surface area of bold seeds will make them prone to mechanical damage, increased infestations of pest and diseases under storage conditions [65, 66]. In addition, it was observed that the black seed coat color and thick seed coat impart higher seed viability under storage [67–69]. Notably, the black soybean seeds are characterized by thicker and tougher testas and slower imbibition than non-black seeds [70]. Studies have shown seed viability is also influenced by seed permeability [64]. Genetic variability and recombination rate within the mapping population for seed size, seed coat color, seed coat thickness and content, testa composition and color, and seed permeability play a crucial role in the identification of closely linked marker and/or location of genes responsible for all the seed longevity related traits. In this study, the RILs showed diversity for their seed and seed coat characteristics. A wide variation was observed in seed dimensions (length, width, and thickness) ranging from smaller to bold sized seeds. The classification of seeds based on the seed coat color of RILs suggested that black seeded lines had higher seed coat percentage and less water permeability as evidenced in the earlier studies [25], thus, regulating the seed germination and seedling vigour through imbibition [71–73]. A similar observation was reported by Kumar et al. [64] in soybean RIL population. A study by Zhang et al. [74] observed that the black and brown seeded F_3_ lines derived from a cross between *G. soja* and *G. max* retained approximately 30% viability after 240 days of soil burial, compared to significantly lower viability in yellow and green seeded lines that evidenced the potential of black seed coat color in maintaining the seed viability under prolonged storage. The association between seed coat color and seed longevity is very well established in soybean with black or dark seed coat color associated with higher longevity [25–27]. In this study, the RIL population broadly exhibited four seed coat colors ranging from yellow to black displaying substantial variability in different seed and seed coat traits. We have also observed variation within the line for depth of coat colour, hence the genetic interpretation will be carried out in the future after developing complete homozygous lines. Earlier studies indicated monogenic [75–78], digenic [77, 79–81], trigenic [71, 80, 82] and complex inheritance [82] for seed coat color. Recombination among specific seed traits is fundamentally important in soybean breeding for improving agronomic qualities and seed longevity. Yellow seed coat is commercially desirable; black seed coat colour is also gaining attention due to nutritional and other medicinal uses. The mean and range for different seed traits for black and yellow groups of RILs showed that many black seed coat genotypes are bold seeded with higher test weight and vice versa. The black and yellow groups differed significantly for water permeability and seed coat percentage. However, the range within each class showed that there are yellow genotypes with low water permeability, indirectly suggesting yellow genotypes with higher seed longevity. Such lines will be useful in commercial breeding. It is important to test the selected genotypes for seed longevity under both natural and artificial ageing techniques before using them in breeding.

Seed coat is the primary point of contact between seed and its environment. Thus, seed coat thickness plays a major role in seed survival [83]. Studies suggest that thicker seed coat confer increased resistance to mechanical damage and pathogen pressure in storage and soil [84]. A positive association was established between seed coat thickness and seed longevity, exhibiting reduced seed mortality in thicker seeds [67, 83]. In this study, correlation analysis between water permeability and seed coat thickness revealed a negative association on both the top and bottom sides of the seed [85]. Our study involving 20 selected RILs with high and low permeability demonstrated that seed coat thickness influence permeability. The seed coat permeability is the major factor in seed viability and longevity [86].

The RILs also showed segregation for hilum color and seed coat lustre. Black seed coat color was dominant with more frequency among RILs. Majority of the seeds possessed black hilum color and intermediate seed coat lustre. Genotypes exhibiting a shiny or smooth luster typically have minimal surface deposits of endocarp, whereas those with a bloom lustre display dense, continuous endocarp deposits arranged in a characteristic honeycomb pattern [86]. It was reported that the seed coat surfaces of the black and brown seeded F_3_ lines were heavily covered with deposited materials forming a honeycomb-like structure with no surface pits, unlike the yellow and green seeded lines, which showed fewer deposits and a flat pitted surface [74]. The seed coat shininess also has been associated with reduced seed imbibition and provides insect and pathogen resistance to common beans [87], thereby improving seed storability. On the other hand, the association of seed hilum color with seed longevity is not established so far. The present study offers to study the relationship between hilum colour, seed coat lustre, and seed longevity.

Overall, RILs population showed segregation for many seed coat characteristics, seed coat thickness and water permeability which are important traits for seed viability. The frequency distribution of all the seven quantitative seed traits showed continuous and normal distribution and appearance of transgressive segregants: thus, consistent with the genetic principle of QTL mapping [56, 88]. The present mapping population capture higher phenotypic variability of the target traits related seed viability in soybean enabling QTL mapping and marker development for molecular breeding. However, the present results are based on only 150 RILs as a representation of 352 lines and are also not completely homozygous. To address the limitations of the study, we are advancing all the 352 lines to complete homozygosity; as a resource for QTL mapping to identify closely linked markers and the genetic analysis of seed traits for higher seed viability.

## Conclusion

The biparental F_5_ segregating mapping population showed extensive variability for all the seed longevity associated traits. The frequency distribution of all the seven quantitative seed traits showed continuous normal distribution with appearance of transgressive segregants: thus, suitable for mapping seed viability and studying seed related traits in soybean. The results also showed that the black seed coat associated with thicker seed coat and low water permeability. Higher range was observed for water permeability and seed coat percentage among the yellow genotypes suggesting the potentiality of the population in identification of recombinants with yellow seed coat colour, low water permeability with higher seed viability. The large mapping population developed in this study has the potential to offer insights into the genetic determinants of seed longevity and form a valuable permanent resource for mapping and/or fine mapping QTLs for seed longevity traits.

## Data Availability

All the generated or analysed data during the study are included in this article. The F_5_ generation RILs are being advanced to succeeding generations using SSD to achieve complete homozygous lines by the first author.
